# Crystal structure of DRIK1, a stress-responsive receptor-like pseudokinase, reveals the molecular basis for the absence of ATP binding

**DOI:** 10.1186/s12870-020-2328-3

**Published:** 2020-04-15

**Authors:** Bruno Aquino, Viviane C. H. da Silva, Katlin B. Massirer, Paulo Arruda

**Affiliations:** 1 Structural Genomics Consortium, Universidade Estadual de Campinas – UNICAMP, Campinas, SP 13083-886 Brazil; 2 Joint Research Center for Genomic Applied to Climate Change (UMIP-GenClima), Campinas, SP 13083-875 Brazil; 3 Centro de Biologia Molecular e Engenharia Genética, Universidade Estadual de Campinas (UNICAMP), Campinas, SP 13083-875 Brazil; 4 Departamento de Genética e Evolução, Instituto de Biologia, Universidade Estadual de Campinas (UNICAMP), Campinas, SP 13083-970 Brazil

**Keywords:** Protein kinase, Pseudokinase, Drought stress, Biotic stress, Abiotic stress

## Abstract

**Background:**

Plants reprogram metabolism and development to rapidly adapt to biotic and abiotic stress. Protein kinases play a significant role in this process by phosphorylating protein substrates that activate or inactivate signaling cascades that regulate cellular and metabolic adaptations. Despite their importance in plant biology, a notably small fraction of the plant kinomes has been studied to date.

**Results:**

In this report, we describe *Zm*DRIK1, a stress-responsive receptor-like pseudokinase whose expression is downregulated under water restriction. We show the structural features and molecular basis of the absence of ATP binding exhibited by *Zm*DRIK1. The *Zm*DRIK1 kinase domain lacks conserved amino acids that are essential for phosphorylation activity. The crystal structure of the *Zm*DRIK1 kinase domain revealed the presence of a spine formed by the side chain of the triad Leu^240^, Tyr^363^, and Leu^375^ that occludes the ATP binding pocket. Although *Zm*DRIK1 is unable to bind nucleotides, it does bind the small molecule ENMD-2076 which, in a cocrystal structure, revealed the potential to serve as a *Zm*DRIK1 inhibitor.

**Conclusion:**

*Zm*DRIK1 is a novel receptor-like pseudokinase responsive to biotic and abiotic stress. The absence of ATP binding and consequently, the absence of phosphorylation activity, was proven by the crystal structure of the apo form of the protein kinase domain. The expression profiling of the gene encoding *Zm*DRIK1 suggests this kinase may play a role in downregulating the expression of stress responsive genes that are not necessary under normal conditions. Under biotic and abiotic stress, *Zm*DRIK1 is down-regulated to release the expression of these stress-responsive genes.

## Background

Plants are sessile organisms that must constantly reprogram their metabolisms to adapt to environmental changes, such as high/low temperature, high/low light intensity, and water restriction. When stress is prolonged for a few days, plant growth and development are affected, leading to yield loss. The responses to those environmental changes are coordinated at transcriptional, translational and posttranslational levels. One of the significant posttranslational modifications occurring is the protein phosphorylation of specific substrates, which is exerted by protein kinases whose expanded families in plants may have significantly contributed to stress adaptation [1, 2]. One class of protein kinases, the receptor-like kinases (RLKs), play significant roles in the stress response in plants. Typically, RLKs have an extracellular domain that generally binds ligands to activate the intracellular kinase domains and consequently modulates signaling pathways by transferring the gamma-phosphate group of ATP to a protein substrate [3, 4]. RLKs comprise the largest group of plant kinases belonging to a distinct family RLK/Pelle, mostly having a leucine-rich repeat (LRR) corresponding to RLK/Pelle LRR subfamilies. The RLK/Pelle family is comprised of more than 600 members out of 1000 kinases in Arabidopsis, more than 1100 members out of 1400 kinases in rice and approximately 760 members out of 1241 kinases in maize [5–7]. Despite the importance and representativeness of RLKs in plants, only a few RLKs have well-known functions [8–10], and fewer of them have their specific ligands identified. Those ligands include endogenous proteins, sulfonated peptides, steroid hormones and pathogen-derived peptide elicitors [4, 11].

The known RLKs have been shown to participate in developmental regulation, disease resistance, and stress tolerance in plants. The growth and development can be exemplified by LRR-RLKs that sense growth-promoting brassinosteroids and to regulate cell elongation and division, such as Brassinosteroid Insensitive 1 (BRI1), or sense peptide hormones to regulate root development such as Root Meristem Growth Factor Receptor 1–3 (RGFR1–3), stem cell maintenance in shoot and root such as Clavata 1 (CLV1), Barely Any Meristem 1–3 (BAM1–3), Receptor-like Protein Kinase 2 (RPK2), abscission and cell separation (HAESA and HAESA-LIKE 2 (HSL2), and stomatal patterning (ERECTA and ERECTA-LIKE 1 (ERL1) [4, 12]. Other LRR-RLKs, such as somatic embryogenesis receptor-like kinases (SERKs) receptor family, including BRI1-Associated Receptor Kinase 1 (BAK1), are involved in a wide spectrum of biological processes, including plant development and disease resistance [13]. Additionally, the RLKs involved in the plant immune system such as Flagellin-Sensitive 2 (FLS2) that senses bacterial flagellin [14], rice Xa-21 conferring resistance to bacterial pathogens [15], and NSP-Interacting Kinase 1 (NIK1) as a defense receptor of geminivirus and begomovirus [16]. The abiotic stress responses driven by RLKs include the abscisic acid (ABA) response, calcium signaling and antioxidant defense against drought, salt, cold, toxic metals/metalloids, ozone and UV-B radiation, and other stresses [2]. Drought stress is an important cause of productivity loss and few RLKs have already being identified as drought sensing receptors. One example is Floral Organ Number 1 (FON1) an ABA sensitivity rice LRR-RLK has been shown to be associated with increased drought tolerance [17]. Other examples are Leaf Panicle 2 (LP2) in rice and Leaf Rust 10 Disease-Resistance Locus Receptor-Like Protein Kinase-Like 1.2 (LRK10L1.2) in wheat that has been associated with drought stress response via ABA-mediated signaling [18, 19]. In an ABA-independent pathway, Stress-Induced Protein Kinase 1 (OsSIK1) [20] and Stress-Induced Protein Kinase 2 (OsSIK2) [21] has been shown to control water loss by stomatal development regulation and upregulation of genes related to detoxification of reactive oxygen species. Another example is BRI1-Like Receptor Kinase 3 (BRL3 recently described as conferring drought tolerance [22].

Approximately 10–20% of eukaryotic kinome members lack phosphorylation activity and are called pseudokinases [10, 23]. In general, those kinases lack essential amino acids for catalysis and thus are not able to phosphorylate any substrate. Regardless of that deficiency, pseudokinases have essential roles in the cell metabolism, acting as kinase scaffolds or as allosteric modulators of signaling components [24–28]. Pseudokinases can act as auxiliary proteins perturbing the conformation of the protein partner by allosteric regulation or acting as a scaffold recruiting a catalytically active kinase to trigger protein activation via phosphorylation. For example, the Arabidopsis pseudokinases Bak1-Interacting Receptor-Like Kinase2 (BIR2), Coryne (CRN) and Receptor Dead Kinase 1 mediate immune responses, stem cell homeostasis, and plant responses to ABA during seedling development, respectively [29–33]. Another example is the receptor-like pseudokinase Guard Cell Hydrogen Peroxide-Resistant 1 (GHR1), which acts as a scaffold interacting with Slow Anion Channel 1 (SLAC1) and with Calcium-Dependent Protein Kinase 3 (CPK3) to induce stomatal closure [26]. In maize, the RLK Pangloss 2 (PAN2), a pseudokinase homologous to GHR1, promotes the polarization of subsidiary mother cell division towards the adjacent guard mother cell during stomatal development [34, 35]. These catalytically inactive kinases retain a high degree of sequence conservation in the kinase domain, suggesting that kinase domain fold and structure are required for signaling activity, while their different biological functions are driven by divergences in the extracellular domains [12, 36, 37]. Nevertheless, it has recently been shown that the specificity of some pseudokinases can also be determined by their intracellular kinase domains, while the ectodomains allow their binding to other transmembrane proteins [4, 26, 38, 39].

Despite the importance of pseudokinases, they have not been well-characterized from the structural biology perspective in plants, which has contributed to a significant knowledge gap to elucidate their functional mechanisms. In this study, we identified a maize RLK that is regulated by water availability from a transcriptional profiling data set [40]. We named this RLK DROUGHT RESPONSIVE INACTIVE KINASE 1, or *Zm*DRIK1. The crystal structure of the *Zm*DRIK1 kinase domain was resolved at the apo form and as a cocrystal structure with a high-affinity small molecule. The mechanism of the absence of ATP binding was elucidated, as well as the transcriptional profiling under water availability characterized. Taken together, the results may help to elucidate the molecular mechanism of the drought stress response and thus help to generate maize lines that are more tolerant to drought stress.

## Results

### DRIK1 is conserved among evolutionarily distant plants

**Fig. 1:**
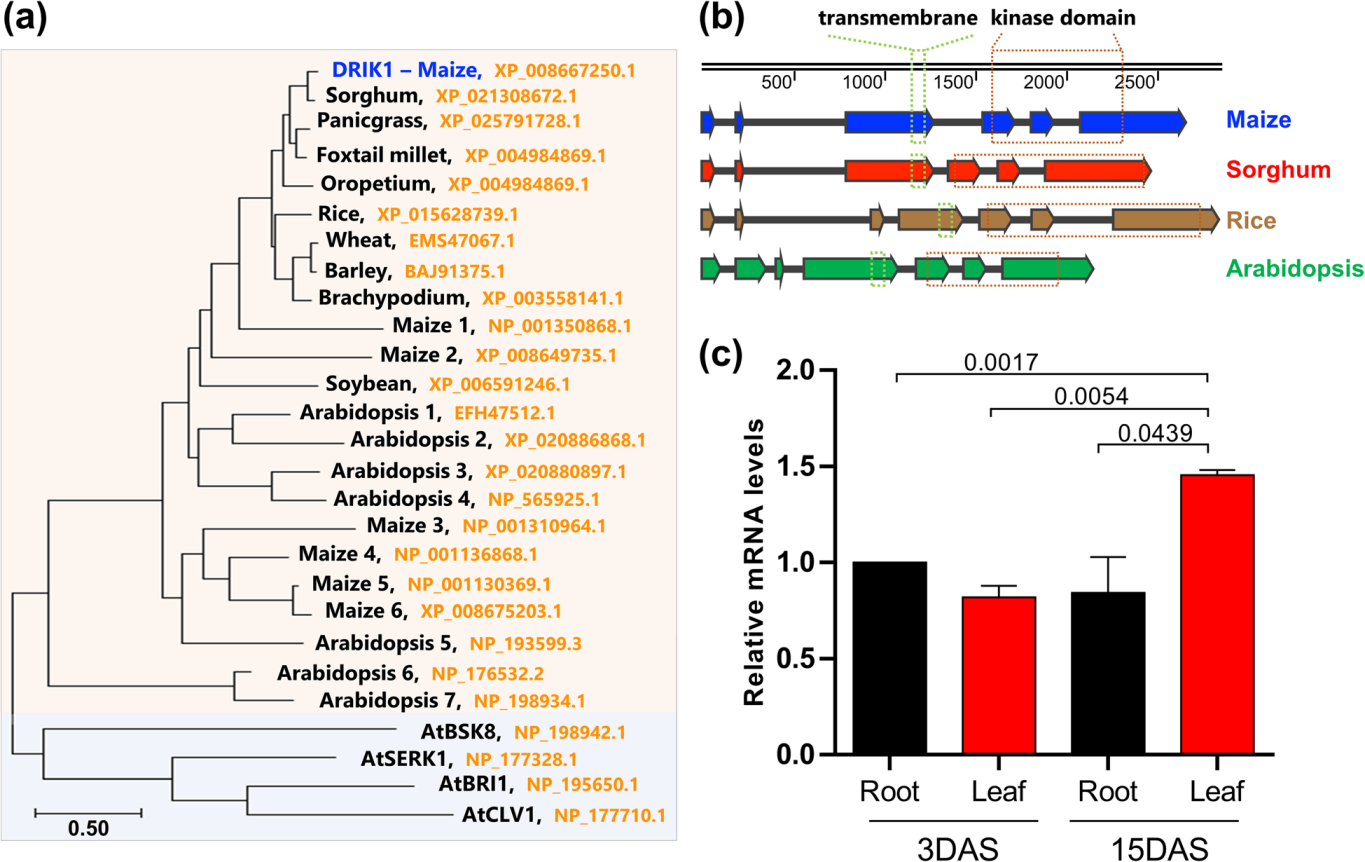
Maize DRIK1 is a conserved RLK expressed in roots and leaves. **a** Phylogenetic analysis of *Zm*DRIK1. Amino acid sequences of representative proteins of the subfamily LRR-VI-2 from several species were aligned, and a phylogenic tree was constructed using the out-groups AtSERK1, AtBRI1, AtCLV1 (catalytically active kinases), and AtBSK8 (catalytically inactive kinase). **b** Intron/exon structure of the *ZmDRIK1* gene compared with closely related genes from maize, sorghum, rice and Arabidopsis. Exons are colored arrows, and introns are black lines. The transmembrane domain (green dotted line) and the kinase domain (red dotted line) corresponding regions are shown. Numbers indicate nucleotide positions along the gene sequence. **c***Zm*DRIK1 relative mRNA content in roots and leaves at 3 and 15 days after sowing (DAS), as determined by qRT-PCR. β-tubulin was used to normalize samples. At alpha 0.05 level, the relative mRNA level of ZmDRIK1 in leaves at 15 DAS was significantly greater than that level in roots, as well as greater than the mRNA level in leaves and roots from younger seedling (3 DAS). The *p*-values of Student’s t test between tissues or age from seedlings, are indicated above the comparative lines in the figure. Error bars indicate standard deviation between three biological replicates

Inspections into the Genevestigator experimental database [41] allowed the identification of *Zm*DRIK1, a receptor kinase that is downregulated under drought stress and upregulated after rewatering in both drought-tolerant and sensitive maize lines, Han21 and Ye478, respectively (Additional file 1: Figure S1). Analysis of the amino acid sequence at the kinase domain revealed alterations in residues essential for ATP binding and kinase activity, suggesting that the protein could be a pseudokinase. We subsequently investigated if the gene encoding DRIK1 is conserved in plants. DRIK1 resembling sequences were retrieved from public databases and inspected for their phylogenetic distribution. DRIK1 homologs were identified in sorghum, rice, wheat, Arabidopsis and soybean (Fig. 1a). Since kinase domains are highly conserved even among kinases from different kingdoms, we analyzed the intron/exon of *DRIK1* gene structures from maize, sorghum, rice and Arabidopsis (Fig. 1b). In maize and sorghum, the closest related analyzed plants, the gene structure is composed of six exons, each having a similar length among the two species. The rice *DRIK1* gene has one small extra exon that appears to be generated by the introduction of an intron in the third exon compared with the *DRIK1* gene from maize. The Arabidopsis *DRIK1* is shorter, but the length of the exons is conserved and more similar to that of rice.

DRIK1 belongs to the LRR VI-2 kinase subfamily, and inspections of this group revealed that the maize *DRIK1* gene is not duplicated (Additional file 2: Figure S2a). The *DRIK1* gene sequence showed low identity when aligned with other maize LRR VI-2 subfamily members, although the region encoding the corresponding kinase domain is highly conserved (Additional file 2: Figure S2b and S2c). In general, plant gene families arose from duplication events during evolution and are paralogous generally having redundant functions. The fact that the *DRIK1* gene is present in a single copy in maize may facilitate further functional analysis to determine its role in plant metabolism, especially the molecular mechanism associated with drought stress response.

The gene expression profile of roots and leaves during the early stages of plant development shows that DRIK1 expression in the leaves is 50% higher in the 15- as compared to that of 3-day-after-sowing (DAS). This result suggests a developmental role of DRIK1 as plant growth and leaf expansion (Fig. 1c).

### *Zm*DRIK1 is regulated by biotic and abiotic stress

Investigations of the RNAseq data from the Genevestigator database revealed that the *ZmDRIK1* gene is downregulated in response to biotic and abiotic stress (Additional file 3: Figure S3). *ZmDRIK1* gene expression is downregulated after infection for 48 h with *Glomerella graminicola* and *Cercospora zeina* fungi. The gene is also downregulated when plants are exposed to high and low temperatures, submergence and drought stress. On the other hand, the gene was upregulated during germination (Additional file 4: Figure S4). However, we must consider these data with caution because they were generated in high-throughput modes that were not specifically validated for *ZmDRIK1*.

**Fig. 2:**
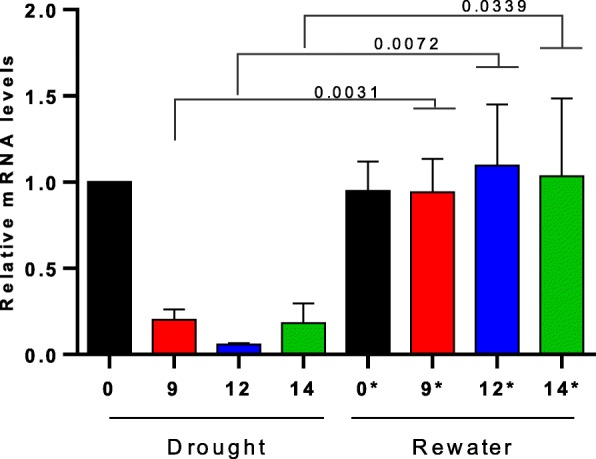
*Zm*DRIK1 expression is downregulated under drought stress. B73 maize plants were grown in pots with soil for 13, 10 or 8 days after which watering was stopped for 9, 12 or 14 days (days of stress), respectively. Half of the stress-treated plants were sampled and immediately immersed in liquid nitrogen and stored at − 80 °C. The other half of the stress-treated plants (marked with *), were rewatered for 24 h and then sampled. Results are average ± standard deviation (three biological replicates) of relative mRNA content of *Zm*DRIK1 in leaves of maize plants, submitted to drought-stress and rewatering treatments. The gene EIF4a was used for expression normalization. At alpha 0.05 level, the relative mRNA level of *Zm*DRIK1 in drought-stressed leaves was significatively lower than its level in leaves of rewatered plants, with the same period of treatment. The *p*-values of Student’s t test between treatments are indicated above the comparative lines in the figure

To experimentally validate the *ZmDRIK1* expression pattern, we analyzed the mRNA level of *Zm*DRIK1 in leaves of plants subjected to drought stress (Fig. 2). Maize B73 plants growing in pots with soil for 8 days had their watering restricted for 9, 12 and 14 days, after which they were rewatered, and leaf samples of both drought-stressed and rewatered plants were analyzed for *Zm*DRIK1 mRNA level. The *Zm*DRIK1 mRNA level was downregulated soon after 9 days of water restriction and was restored after 24 h of rewatering (Fig. 2).

### *Zm*DRIK1 is a receptor-like pseudokinase

**Fig. 3:**
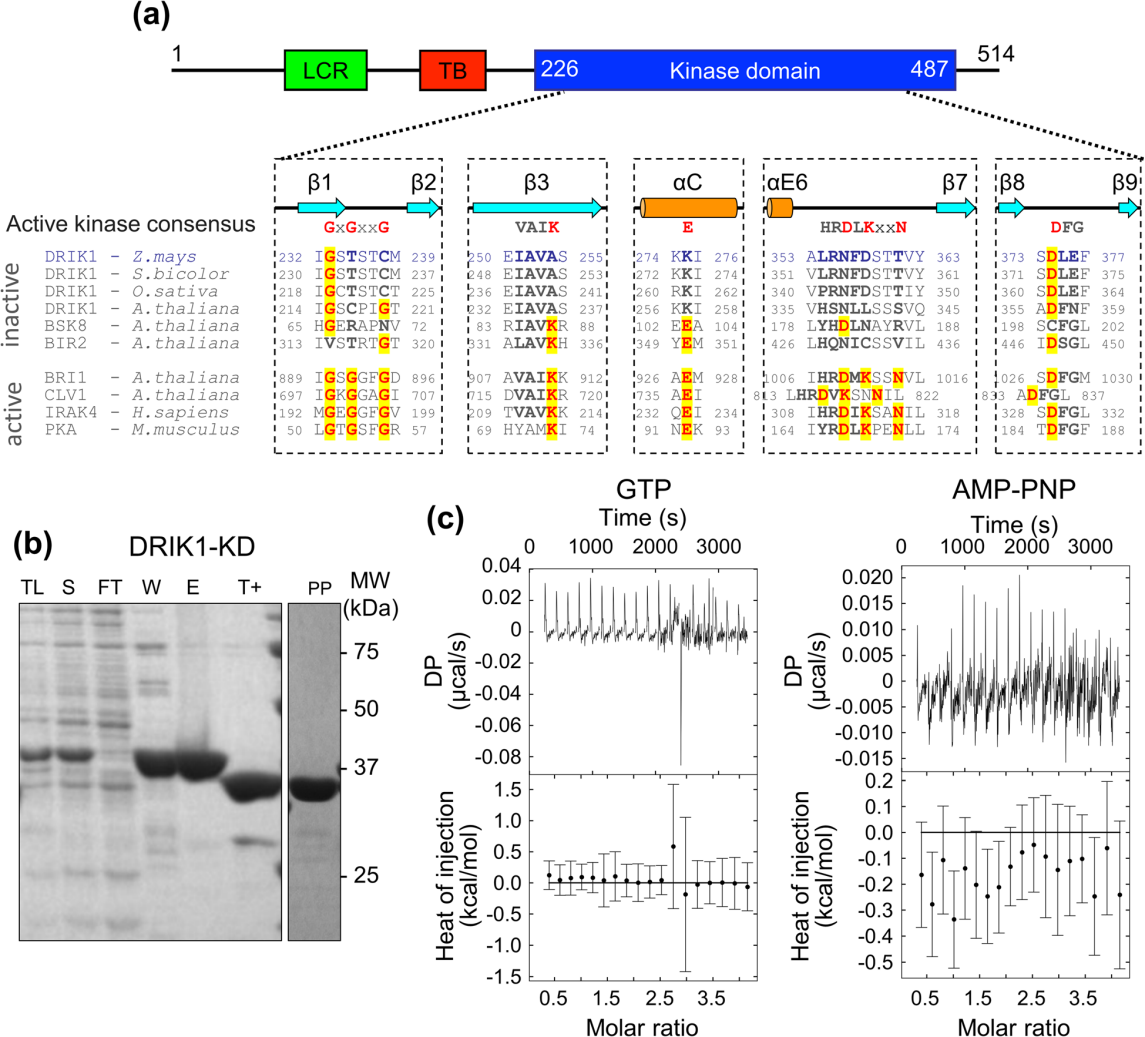
DRIK1 is a pseudokinase that is unable to bind nucleotides. **a***Zm*DRIK1 protein domain structure and multiple amino acid sequence alignment of *Zm*DRIK1 with a set of active and inactive RLK/Pelle-LRRs. The schematic *Zm*DRIK1 domain structure shows the position of the low complexity region (green), the transmembrane domain (red) and the kinase domain (blue). Numbers indicate amino acid positions along the protein sequence. The multiple amino acid sequence alignment shows the core of the kinase domains of: DRIK1 from maize, sorghum, rice and Arabidopsis; BIR2, BRI1 and BSK8 and CLV1 from Arabidopsis; IRAK4 from *Homo sapiens;* PKA from *Mus musculus*. Conserved residues, essential for kinase activity, are marked in red. Highlighted in yellow are the conserved residues representing the consensus kinase sequence. Secondary structures (β-strands in light blue arrow; α-helix in orange cylinders) are named according to the PKA structure, except αE6, which in PKA is a β-strand 6. **b** Purification of the *Zm*DRIK1 kinase domain. *Zm*DRIK1-KD^R187-S514^ was cloned into pNIC28a-Bsa4 and expressed in *E coli* BL21(DE3) pRARE. Lanes are TL, total lysate; S, supernatant; FT, flow through; W, washed out; E, eluted fraction, T+, TEV protease treated. PP, gel filtration purified *Zm*DRIK1-KD^R187-S514^. MW, molecular weight of protein standards in kDa. The original SDS-PAGE gels can be viewed from Additional file 9: Figures S6-S7. **c***Zm*DRIK1-KD^R187-S514^ binding assays for GTP and AMP-PNP as analyzed by ITC

*Zm*DRIK1 is a transmembrane protein with an extracellular domain harboring a low-complexity region and a kinase domain in the intracellular portion of the protein. In general, active kinases have amino acid residues at conserved positions that are essential for kinase activity. To confirm that *Zm*DRIK1 is an inactive kinase, we aligned DRIK1 from different species and with active (BRI1; CLV1; IRAK4; PKA) and inactive (BSK8; BIR2) kinases (Fig. 3a). DRIK1 from maize, sorghum, rice, and Arabidopsis have minimal amino acid sequence variation among them. In the P-loop, a conserved sequence Gly-X-Gly-X-X-Gly is observed in active kinases [42], while in maize, sorghum, and rice, the second and third glycines are substituted by Thr and Cys, respectively. As these changes occurred for small amino acids, they might not influence ATP binding or even the catalytic activity of DRIK1. Another important feature of active kinases is the conserved Asp-Phe-Gly motif, where Asp is important to coordinate the Mg^2+^ ion with ATP. In DRIK1, the Asp-Phe-Gly motif is changed to Asp-Leu-Glu, and the conservation of the amino acid Asp suggests that this feature is not compromised. On the other hand, the Asp residue from the His-Arg-Asp-X-Lys-X-X-Asn motif of the catalytic loop is essential to accept the hydrogen from the hydroxyl group being phosphorylated [43]. In DRIK1, this amino acid is substituted by the polar uncharged amino acid Asn, suggesting that phosphorylation could not happen. One of the most important kinase catalytic amino acids is the Lys in the Val-Ala-Ile-Lys motif. In DRIK1, from all species analyzed, this amino acid is substituted by Ala. In addition, the amino acid Glu from the C-helix is substituted by Lys. These two amino acids (Lys from Val-Ala-Ile-Lys motif and Glu from C-helix) form a salt bridge that is important for interacting with ATP in the ATP-binding pocket and promoting phosphorylation. Therefore, these amino acid changes suggest that even if *Zm*DRIK1 was able to bind ATP, the absence of catalytic amino acids strengthens its pseudokinase nature.

Several pseudokinases, despite being unable to phosphorylate other proteins, can bind ATP [44–46] and change conformation to interact with other proteins and regulate metabolic pathways [47, 48]. We investigated whether *Zm*DRIK1-KD could bind nucleotides. The purified recombinant *Zm*DRIK1-KD^R187-S514^ (Fig. 3b) did not bind GTP or the ATP analog AMP-PNP (Fig. 3c).

### Triad Leu240, Tyr363 and Leu375 impairs nucleotide binding at the ATP pocket

**Fig. 4:**
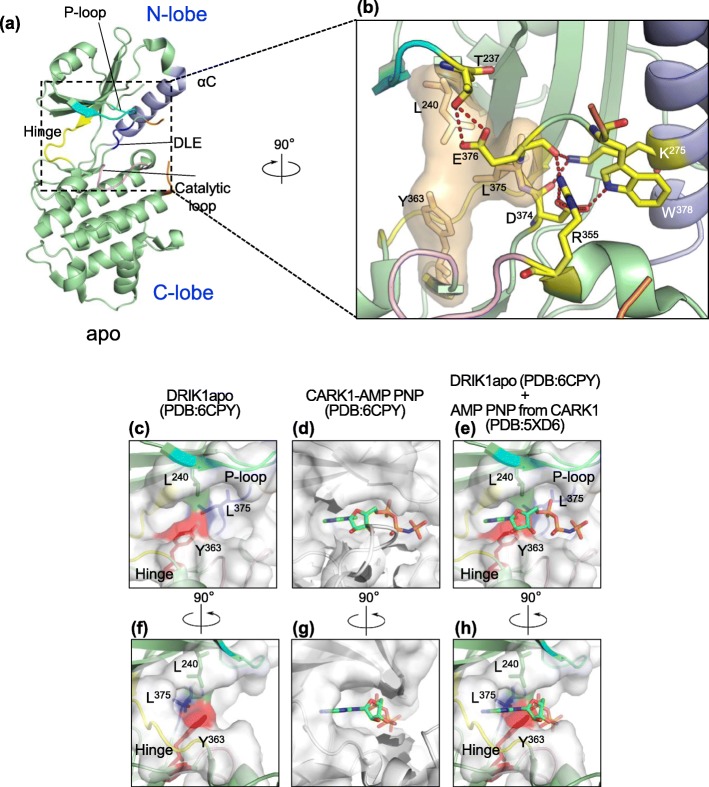
Crystal structure of *Zm*DRIK1 apo form. **a** Cartoon representation of the *Zm*DRIK1-KD structure in the apo form. Structure features: glycine-rich (P-loop – cyan; hinge region – yellow; catalytic loop -pink; DLE (more commonly DFG) motif – blue; activation segment – orange; C-helix – purple. Other protein regions are in green. **b** Closer look at the ATP binding pocket. Amino acids important for ATP binding and pocket stabilization are shown as stick (carbon in yellow; oxygen in red; nitrogen in blue) and the interaction between them in red dashed lines. Surface of amino acids Leu^240^, Tyr^363^ and Leu^375^ are shown in light orange. (**c** and **f**) Catalytic pocket of apo *Zm*DRIK1-KD (PDB: 6CPY). Leu^240^; Tyr^363^ and Leu^375^ are shown as sticks, and the surfaces of these amino acids are colored green, blue and red, respectively. (**d** and **g**) Catalytic pocket of CARK1 cocrystallized with AMP-PNP (PDB: 5XD6). AMP-PNP is represented in stick, and atoms are colored as follows: carbon – green; nitrogen – blue; oxygen - red; phosphate – orange. (**e** and **h**) Superposition of the *Zm*DRIK1 ATP binding site with AMP-PNP from the CARK1 structure. The protein molecular surface is shown in white with 50% transparency

To understand the lack of nucleotide interaction with the *Zm*DRIK1-KD ATP pocket at the molecular level, we solved the *Zm*DRIK1-KD crystal structure at 1.7 Å. The crystal structure of the protein spanning amino acids Leu^209^ to Pro^491^ revealed a canonical kinase domain structure with an N-terminus lobe, mainly formed by β-sheets, and a C-terminus lobe, mainly formed by α-helix, that are connected by a hinge (Fig. 4a). The protein portion spanning amino acids 380 to 394 of the activation loop and amino acids 419 to 425 of the loop formed between F and G-helix in the C-terminus lobe were not observed in the density map, probably because of the flexibility of these regions.

The ATP binding pocket is structured as a cavity exposed to the solvent (Fig. 4b and e). However, the side chain of the Leu^240^, Tyr^363^ and Leu^375^ amino acids triad occupies the ATP binding site, suggesting that this conformation might block ATP accommodation in the pocket. Leu^240^ belongs to the β2-strand and interacts with the β1-strand through the atoms from the main chain. This interaction stabilizes and exposes the side chain of this amino acid toward the ATP binding pocket. In addition, Leu^375^, which belongs to the Asp-Leu-Glu motif (commonly Asp-Phe-Gly), is stably oriented to the ATP binding pocket due to a series of interactions involving amino acids from C-helix, activation loop and catalytic loop. In C-helix, Lys^275^, which replaces the conserved Glu, interacts with the backbone of Asp^374^ and Glu^376^ from the Asp-Leu-Glu motif, supporting a conformation that induces the leu^375^ from the Asp-Leu-Glu motif side chain to face the ATP binding pocket. Hence, this conformation is stabilized by the interaction of conserved Arg^355^ (from the His-Arg-Asp-X-Lys-X-X-Asn motif) from the catalytic loop with the main chain of Glu^376^ and by the interaction between the side chain of Trp^378^ from the activation loop and Asp^374^ from the Asp-Leu-Glu motif. Together, these interactions force the side chain of Leu^375^ to be positioned towards the ATP binding pocket. Finally, the position of the side chain of Tyr^363^ is stabilized by Lys^371^ from the β7-strand and Gly^312^ from the hinge. Interaction energy matrix (IEM) [49] analysis revealed that the side chains of these three amino acids also interact with favorable interaction energy (Tyr^363^-Leu^375^: − 5.75 kJ/mol; Tyr^363^-Leu^240^: − 1.26 kJ/mol; Leu^240^-Leu^375^: − 5.49 kJ/mol).

To further elucidate the role of Leu^240^, Tyr^363^ and Leu^375^ in preventing ATP binding, we superimposed the apo structure of *Zm*DRIK1-KD (Fig. 4c and f) with AMP-PNP from the cocrystal structure of Cytosolic Aba Receptor Kinase 1 (CARK1) (Fig. 4d and g) [50]. It is possible to observe in the image superimposition (Fig. 4e and h) that in the ATP-binding pocket of *Zm*DRIK1-KD, there is enough space to accommodate the nitrogen base of ATP between the hinge and the gatekeeper. The phosphate portion of ATP also does not have any obvious limitations in interacting with the *Zm*DRIK1-KD ATP-binding pocket. However, the side chains of Leu^240^, Tyr^363^ and Leu^375^ are oriented to the ATP-binding pocket, forming a barrier in the middle of the ATP binding pocket. In the superposition of AMP-PNP from CARK1 and *Zm*DRIK1-KD, it is possible that the sugar portion of ATP might be impaired by steric hindrance. Thus, this spine formed by the side chain of these three amino acids occludes the ATP binding pocket of *Zm*DRIK1-KD, which might explain why nucleotide is unable to bind this kinase.

### The ATP pocket of *Zm*DRIK1-KD binds the ENMD-2076 small molecule

**Fig. 5:**
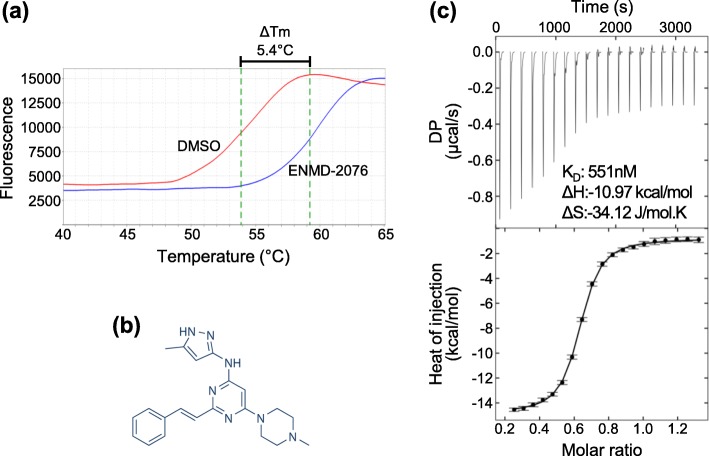
*Zm*DRIK1-KD binds the small molecule ENMD-2076. **a** Thermal shift assay of ENMD-2076 binding to *Zm*DRIK1-KD^R187-S514^, as determined by DSF. The compound stabilization is shown by the dislocation of the protein thermal denaturation midpoint (Tm) in the presence of ligand versus in DMSO only (red line). **b** Chemical structure of the ENMD-2076 molecule. **c** Determination of the thermal dynamic properties of the ENMD-2076 interaction with *Zm*DRIK1-KD^R187-S514^ by ITC

Although *Zm*DRIK1-KD is not able to bind nucleotides, other molecules might interact with its ATP binding pocket and regulate its activity. Using a DSF assay (Fig. 5a; Additional file 5: Figure S5) we screened a small molecule library of 378 compounds designed for human kinases and identified the ENMD-2076 compound (Fig. 5b). ENMD-2076 bind *Zm*DRIK1-KD with a ΔTm shift higher than 3.5 °C, which suggests that this molecule can thermally stabilize the kinase domain (Additional file 6: Table S1). The interaction of ENMD-2076 with *Zm*DRIK1-KD^R187-S514^ was further confirmed by ITC with a K_D_ of 551 nM, indicating a high affinity binding mode (Fig. 5c).

**Fig. 6:**
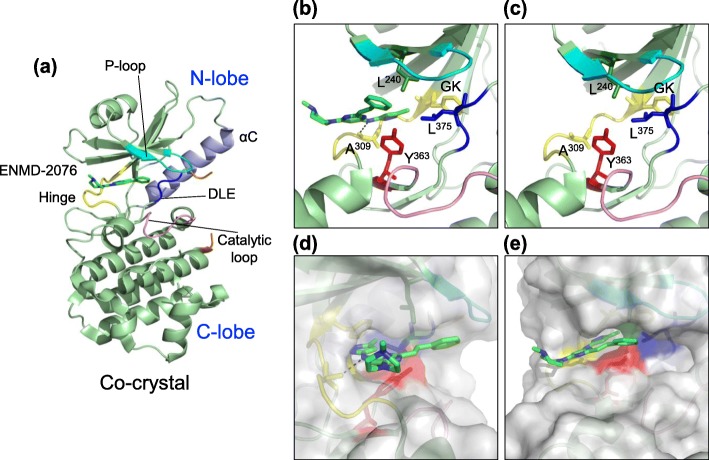
Cocrystal structure of *Zm*DRIK1-KD bound with ENMD-2076 at the ATP binding pocket. **a** Cartoon representation of the cocrystal structure of *Zm*DRIK1-KD bound with ENMD-2076. Protein features are colored as follows: glycine-rich (P-)loop - cyan; hinge region - yellow; catalytic loop -pink; DLE (more commonly DFG) motif - blue; activation segment - orange; C-helix – purple. Other protein regions are in green. ENMD-2076 is shown in stick and colored as follows: carbon – green; nitrogen – blue. **b** and **c** Structural features of the *Zm*DRIK1-KD ATP binding pocket bound with ENMD-2076 (**b**) and apo form (**c**). Residues Leu^240^, Tyr^363^, Leu^375^, ENMD-2076 and the gatekeeper (Phe) are represented in sticks. The black dashed line (**b**) indicates an interaction between protein and ENMD-2076. **d** and **e** Surface representation of the cocrystal *Zm*DRIK1-KD-ENMD-2076

DSF and ITC can be used to identify new ligands and elucidate the physical properties of the interaction. However, these assays do not show the interaction of the molecule with the protein at the molecular level. To gain insight into how *Zm*DRIK1 can bind to an ATP-competitive inhibitor, we solved the cocrystal structure of *Zm*DRIK1-KD bound to ENMD-2076. In this cocrystal structure, it is possible to observe that ENMD-2076 binds the ATP binding pocket and that its amine group from pyrimidine acts as a hydrogen donor for the Ala^309^ carbonyl from the hinge (Fig. 6b-dashed black line). When we compared the ATP-binding pocket of the *Zm*DRIK1-KD apo form (Fig. 6c) with the cocrystal (Fig. 6b), we observed no major changes induced by the small molecule ligand, especially in the hinge and the ATP binding pocket.

The piperazine from ENMD-2076 fits snugly in the cavity expected to be fulfilled by the nitrogen base of the ATP. The pyrazole is exposed to the solvent, and this group may be a target for modifications without compromising the interaction of ENMD-2076 with *Zm*DRIK1-KD. The styrene is oriented to the cavity believed to be occupied by the phosphates from ATP. Unlike ATP, the side chain of the triad Leu^240^, Tyr^363^ and Leu^375^ did not impair the interaction with ENMD-2076.

## Discussion

The analysis of global expression patterns in an organism using the transcriptome helps elucidate how metabolism adapts in response to stress. However, transcriptome experiments do not demonstrate the role of individual proteins in normal or stressed metabolism. Nevertheless, using public databases to identify genes that have an expression pattern regulated by metabolic perturbations can be the starting point to elucidate their role. In this work, we identified *Zm*DRIK1, a conserved receptor-like pseudokinase that is regulated by biotic and abiotic stress.

Pseudokinases have emerged as important signaling players that regulate cell metabolism by recruiting other proteins and thereby activating a responsive pathway [23, 36]. In Arabidopsis, the interaction between Clavata2 (CLV2), a receptor-like protein (LRR-RLP), and Coryne, a receptor-like pseudokinase, is important for stem cell maintenance in shoots [32]. Despite a lack of transferase activity, the kinase domain of Coryne is necessary to promote the endoplasmic reticulum-to-plasma membrane migration of CLV2, indicating that even without phosphorylation activity, these kinases have important roles in plant metabolism. In maize, stomata formation is dependent on two other receptor-like pseudokinases, PAN1 and PAN2 [51, 52]. These two pseudokinases participate in a series of events that polarize first PAN2 and then PAN1 to promote asymmetric cell division of a precursor subsidiary mother cell generating functional stomata [53]. In *pan1*- or *pan2*- knockouts, the subsidiary mother cells did not undergo asymmetric cell division; thus, malformed stomata are produced, implicating in nonfunctional stomata. Moreover, pseudokinases also have roles in the response to biotic and abiotic stress. In response to biotic stress in Arabidopsis, a cytoplasmic pseudokinase named HOPZ-ETI-DEFICIENT 1 (ZED1), is essential to respond to *Pseudomonas syringea* infection [54]. This pseudokinase serves as a decoy to HopZ1a, an infection effector, in the ZAR1-mediated immunity response. Another pseudokinase important to respond to abiotic stress is GHR1. This LRR-RLK is necessary to activate SLAC1 and promote stomatal closure [26]. It was proposed that this pseudokinase don’t phosphorylate SLAC1 but serve as a scaffold for additional regulators, such as CPK3. The widespread relevance of plant pseudokinases must become even more complex as more proteins are characterized. In particular, considering the increasing number of human pseudokinases involved in a variety of functions, binding partners, conformations and molecular mechanisms of action [25]. In this study, we identified the pseudokinase *Zm*DRIK1 that is phylogenetically, genetically structurally conserved among different plant species but less conserved among the other representative members of the same subfamily LRR-VI-2. The conserved gene structure in maize, sorghum and rice suggests a similar function in these plants, as well as a common ancestor during evolution [55]. Although the tandem duplication of a few subfamilies of RLK/Pelle has been described [8], the missed paralogous relationship between *Zm*DRIK1 and other members of the same subfamily was not surprising, as the gene duplications of pseudokinases are believed to be evolved from canonical kinases [56], while all 6 other members of LRR-VI-2 from maize are also predicted to be pseudokinase [10]. In addition, the sequence alignment of maize genes from LRR-VI-2 showed very low similarity with *Zm*DRIK1, while their kinase domains are highly conserved, corroborating the remarkable aspect of both known and putative dead kinases retaining selected constraints of conserved kinase domain sequences [10] and divergences in extracellular domains [4, 6]. Little is known about the molecular function of pseudokinases in general but particularly from the LRR-VI subfamily [10, 57]. Among these pseudokinases, only two members were previously characterized in Arabidopsis, MDIS1 and MDIS2. The MDIS1/Male discover 1 was found to form heterodimers with receptors MDIS1-Interacting Receptor Kinases MIK1 and MIK2 on the pollen tube that perceives female attractant LURE1 [58]. Although predicted as a pseudokinase, MDIS1 can be phosphorylated by MIK1 [58], acting as a putative coreceptor [59]. The MDS2 (MRH1, [60]) was described as being associated with root hair formation and interacting with the plant potassium channel AKT2 [60, 61]. Our phylogenetic and gene structure analyses revealed that DRIK1 is probably functionally distinct from these two previously characterized proteins.

Pseudokinases are characterized by the absence of conserved amino acids for kinase activity; despite compromised phosphorylation, several of these proteins retain the ability to bind ATP [62]. On the other hand, some catalytically active RLKs have dispensable kinase domains, depending on the pathway-specific factor interaction [32, 63, 64]. In plants, BSK8, a pseudokinase involved in brassinosteroid signaling, binds to ATP in an unusual conformation, and the complex protein:nucleotide might regulate other proteins [46]. However, for *Zm*DRIK1, ATP binding was not observed. Similarly, the pseudokinase BIR2, a negative regulator of the BAK1-mediated defense mechanism, also doesn’t bind to ATP; in this case, the unusual position of the P-loop forms a barrier that occludes the ATP binding site [29]. Although the ATP binding pocket is exposed to the solvent in *Zm*DRIK1, the P-loop doesn’t show the consensus sequence common to active kinase, and the interaction between Thr^237^ and Glu^376^ (from Asp-Leu-Glu motif) suggests that this structure is more rigid and may compromise ATP exchange, as observed by Kwon et al. (2019) [10]. Moreover, the conserved Asp-Phe-Gly motif and Glu from C-helix which, in active kinases, interact with each other and the catalytic lysine are substituted by Asp-Leu-Glu and Lys, respectively. These mutations cause a rearrangement in the interaction in this core, leading to a stable conformation of the Asp-Leu-Glu motif that position the side chain of Leu^375^ oriented to the ATP binding pocket. Hence, the interaction between the β1-strand and β2-strand also positions the side chain of Leu^240^ in the ATP binding pocket, and Tyr^363^ is stabilized by interaction with amino acids from the hinge and β7-strand. Although these three amino acids occupy the ATP binding pocket, this cavity is structured and accessible and might bind to other molecules, such as ENMD-2076. This small ligand, in addition to blocking ATP binding, can also preferentially stabilize *Zm*DRIK1 in a conformational state able to activate or inactivate the protein, similar to that observed in several human kinases and pseudokinases with small-molecule binding [25]. Using the cocrystal structure, these molecules can be rationally designed to elucidate the protein’s role in plant metabolism.

Bioinformatics analysis of the *Zm*DRIK1 expression pattern using publicly available RNA-seq data from maize also suggests a possible role for *Zm*DRIK1 in plant development: the upregulated expression during maize germination and downregulation under a variety of stress conditions suggests the growth regulation in detriment of alert-state status to defense under stress conditions. This mechanism of stress response generally comes at the cost of reduced growth [65, 66]. On the other hand, this gene might act as an inhibitor of stress response. In this case, under normal conditions, this gene kept the stress response pathway inactivated, and when plants must respond to stress, the protein levels decrease, releasing the responsive pathway. Once the stress condition ends, the protein levels increase to inhibit the stress response pathway. Thus, to answer this question, further physiological data will be necessary and are beyond the structural scope of the current study. Genetic modulation *“in plants”* will be an important tool to elucidate if the protein kinase fold devoid of catalytic activity is dispensable for *Zm*DRIK1 function in maize, whereas small molecules, such as ENMD-2076 and improved derivatives could be used for modulation of *Zm*DRIK1 functions through the design of conformation-specific inhibitors.

## Conclusions

*Zm*DRIK1 is a novel receptor-like pseudokinase responsive to biotic and abiotic stress. The absence of ATP binding and consequently, the absence of phosphorylation activity, was proven by the crystal structure of the apo form of the protein kinase domain. The side chains of amino acid triad Leu^240^, Tyr^363^ and Leu^375^ faces the ATP-binding pocket, forming a barrier. The spine formed by the side chain of these three amino acids occludes the ATP pocket preventing the nucleotide binding.

The expression profiling of the gene encoding *Zm*DRIK1 suggests this kinase may play a role in downregulating the expression of stress responsive genes that are not necessary under normal conditions. However, when plants are subjected to biotic and abiotic stress, *Zm*DRIK1 is down-regulated to release the expression of stress-responsive genes.

## Methods

### Identification of maize kinases down-regulated by drought stress and other perturbations

The Genevestigator database [41] was used to select candidate genes responsive to stress. By analyzing the gene expression of plants subjected to drought treatments, promising targets were selected for further characterization, including Zm00001d028770, which encodes *Zm*DRIK1. The publicly available maize microarray data from Zheng et al. 2010 [40] was chosen to investigate RLKs showing downregulation under drought and upregulation under rewatering treatments in Han21 and Ye478 drought-tolerant and drought-sensitive maize lines, respectively. As one of the top 10 candidates, *ZmDRIK1* showed differential expression log-ratio > 2 in all 4 samples (both genotypes and treatment). All other transcriptional expression profiles of *ZmDRIK1* under different perturbation conditions were analyzed from experimental data available in the Genevestigator database. The experimental data were retrieved from Genevestigator, analyzed and compiled using GraphPad Prism version 6.01 for Windows (GraphPad Software, La Jolla, USA). Unless otherwise specified, all the expression data were from the mRNA-Seq platform and were from the B73 inbred line.

### Plant material and growth conditions

Maize (*Zea mays*) inbred line B73 were obtained from Maize Genetics Cooperation Stock Center (http://maizecoop.cropsci.uiuc.edu). Seeds were surface sterilized by immersion in 15% sodium hypochlorite solution containing 0.1% Tween 20 for 15 min followed by eight washes in distilled water. The seeds were enrolled in paper towel and incubated at 28 °C for 72 h, and the seedlings were subsequently harvested or transplanted to pots containing a mixture of soil and vermiculite 1:1 (w/w) and grown for 15 days in a growth chamber at 28 °C and 16/8 h day/night. Leaves and roots were sampled and immediately frozen in liquid nitrogen before being used for qPCR analysis. For the drought stress experiments, seedlings were grown in pots containing a mixture of sphagnum:perlite (7:1 v/v) supplied with PG Mix YARA 14–16-18 under controlled conditions of 25 °C for 16 h light and ~ 45% relative humidity. Progressive drought stress was introduced 8 days after sowing by irrigation restriction for 9, 12 or 14 days, while control plants were well-watered. Leaves were harvested, immediately frozen in liquid nitrogen, and stored at − 80 °C before use.

### Cloning, expression and purification of *Zm*DRIK1 kinase domain (*Zm*DRIK1-KD*)*

cDNA from the maize B73 inbreed line prepared from total RNA isolated from young leaves was used as a template for the amplification of a DNA fragment encoding the *Zm*DRIK1 kinase domain. Two forward primers (drik1-Arg^187^-F - TACTTCCAATCCATGCGAGCTAAGAAAATGGGAACCG); drik1-Lys^217^-F - TACTTCCAATCCATGAAACGATCAGAGCTGGAAACGG) and two reverse primers (drik1-Ser^514^-R - TATCCACCTTTACTGTCAGCTCTCAGAAGTCATAATCTCAAGC); drik1-Pro^488^-R - TATCCACCTTTACTGTCAAGGCCCTAGCGCGG) were designed. The combination of these primers generated four constructs spanning the *Zm*DRIK1 kinase domain (residues Arg^187^-Ser^514^; Lys^217^-Ser^514^; Arg^187^-Pro^492^; Lys^217^-Pro^492^). Amplicons were treated with T4-DNA polymerase for ligase-independent cloning (LIC) [67] and cloned in pNIC28a-Bsa4 [67]. Confirmation of positive clones was made by colony PCR and sequencing.

Plasmid pNIC28a-Bsa4 harboring each construct was transformed into BL21(DE3)-pRARE, and colonies were inoculated in 30 mL of LB medium and grown overnight in a shaker at 37 °C. Cultures were diluted in 1.5 L of TB medium and grown until OD_600_ reached 1.5–2. Cultures were cooled to 18 °C, and protein synthesis was induced overnight by 0.2 mM IPTG. Bacteria were harvested by centrifugation at 5,000 x g; 10 min; 4 °C, suspended in 2x binding buffer (1x binding buffer is 50 mM HEPES pH 7.4, 500 mM NaCl, 5% glycerol, 10 mM imidazole, and 1 mM TCEP) with 1 mM of PMSF and stored at − 80 °C.

The suspended pellets were thawed, and the cells were lysed by sonication for 12 min (5 s ON; 10 s OFF; Amp 30%). One milliliter of 5% polyethyleneimine was added per 30 ml of lysate, and the lysate was then clarified by centrifugation at 40,000 x g; 45 min; 4 °C. The supernatant was loaded onto an IMAC column (5 ml HisTrap FF Crude, GE Healthcare, Uppsala, Sweden), and contaminants were washed with a binding buffer with 30 mM imidazole. The recombinant protein was eluted with 300 mM imidazole in binding buffer. Eluted protein was treated with TEV protease to remove the 6xHIS-tag. Contaminants and the tag were removed using nickel beads. As the last step of purification, proteins were injected onto a size exclusion HiLoad 16/60 Superdex 200 pg (GE Healthcare, Uppsala, Sweden) column equilibrated with binding buffer without imidazole. Fractions of purified protein were pooled together and stored at − 80 °C.

### Isothermal calorimetry

Isothermal calorimetry (ITC) was used to determine the interaction between *Zm*DRIK1-KD^R187-S514^ with nucleotides and small molecule ligands. For the nucleotide, 50 μM of purified *Zm*DRIK1-KD^R187-S514^ (cell) was titrated with 1 mM GTP or the ATP analog AMP-PNP (injectant) in ITC buffer containing 50 mM K-phosphate pH 7.5, 500 mM NaCl, 5% glycerol, 1 mM TCEP and 5 mM MgCl_2_. For the small molecule ligands, 50 μM of ENMD-2076 (cell) was titrated with 300 μM of *Zm*DRIK1-KD^R187-S514^ (injectant) in ITC buffer without magnesium. The heat of interactions was measured on a MicroCal Auto iTC200 (GE/Malvern Panalytical, Northampton, UK), with titrations at 25 °C in a stirring speed of 750 rpm and 300 s between each 2 μL injection. Dilution heat were measured titrating buffer and ligands alone and then subtracted from protein-ligand data. Protein concentrations used in this experiment were measured using Edelhock method [68]. Data were analyzed using NITPIC, version 1.2.2 and Sedphad, version 12.1b software. Figures were generated in GUSSI version 1.3.2 [69].

### Crystallization, data collection, structure determination and refinement

*Zm*DRIK1-KD was crystallized in the apo form and bound to ENMD-2076. For the apo form, 857 μM of purified *Zm*DRIK1-KD^R187-S514^ was centrifuged for 10 min at 21,130 x g at 4 °C, and a 150-nL-volume drop was pipetted by mixing purified protein and crystallization solution from the JCSG-plus HT96 (Molecular Dimension, Maumee, USA) crystallization screen at three ratios (2:1, 1:1, 1:2). For cocrystallization, purified *Zm*DRIK1-KD^R187-S514^ was incubated with ENMD-2076 at a threefold molar excess for 30 min on ice. Mixtures were centrifuged for 10 min at 21,130 x g at 4 °C, and 150-nL-volume drops were pipetted at three ratios (2:1; 1:1; 1:2) using a custom optimization crystallization screen (Molecular Dimension, Maumee, USA). Crystallization plates were incubated at 20 °C until crystals appeared. Crystals were collected in a cryoprotectant solution (reservoir solution supplemented with 30% glycerol) and flash-frozen in liquid nitrogen. Diffraction data were collected at the Advanced Photon Source (APS) or Diamond Light Source (DLS). Data were processed using first XDS [70] and latter AIMLESS, from CCP4 software suite [71]. Phaser [72, 73] was used to perform the molecular replacement with the kinase domain of SUCROSE INDUCED RECEPTOR KINASE 1 (SIRK1) (PDB: 5UV4). After the model was built, the refinement of the structure was made using Coot [74] followed by the validation of the structure factors and coordinates using MolProbity [75]. Structure factors and coordinates (Table 1) were deposited in the Protein Data Bank with accession number 6CPY for the apo form and 6EAS for the cocrystal.

**Table 1:** Crystallographic data

Data collection	***Zm***DRIK1-KD apo	***Zm***DRIK1-KD + ENMD-2076
X-ray source	APS BEAMLINE 24-ID-E	DIAMOND BEAMLINE I24
Wavelength (Å)	0.979180	0.976230
Space group	P 21 21 21	P 21 2 21
Cell dimensions (Å) a, b, c (Å)	61.49 61.5144.76	60.51 61.79 65.38
Resolution (Å)	19.82–1.7	28.9–2.0
No. of unique reflections	58,167 (2974)	17,144 (1238)
Rmerge (%)	6.4 (203)	5.5 (87.1)
Mean I/σI	16.4 (1.62)	14.6 (2.0)
Completeness (%)	99.9 (99.9)	99.9 (100)
Redundancy	10.9 (10.7)	6.4 (6.8)
CC ^1^/_2_	0.99 (0.519)	0.99 (0.72)
**Refinement**		
Resolution range (Å)	19.82–1.7	28.9–2.0
R/Rfree (%)	19.9/21.5	20.81/25.08
Mean B-factor (Å)	35.0	48.0
r.m.s.d. bond lengths (Å)	0.0066	0.0072
r.m.s.d. bong angles (degrees)	1.10	1.11
**Ramachandran plot statistics (%)**		
Preferred regions	99.05	99.61
Outlier	0.0	0.0
**PDB ID**	6CYP	6EAS
**Crystallization conditions**	0.15 M potassium bromide, 30% PEG2000 MME	1 mM zinc chloride; 22% PEG6000;100 mM MES; pH 6.0

### Differential scanning fluorimetry (DSF)

A cell-permeable ATP-competitive kinase inhibitor library from Selleckchem (Houston, TX, United States; catalog No. L1200) was screened to identify interactors for *Zm*DRIK1-KD. One micromolar of each purified construct of *Zm*DRIK1-KD was mixed with DSF buffer (100 mM K-phosphate, 150 mM NaCl, 10% glycerol, and pH 7.5) containing 1:1000 SYPRO Orange Protein Thermal Shift Dye (Life Technologies Corporation, Eugene, USA) in a 384-well plate containing 10 μM of each library compound. As compound stocks were stored at 10 mM in 100% DMSO, a control with 0.1% DMSO was used as a reference. Plates were sealed using optically clear films, and fluorescence intensity data were measured in a temperature gradient from 25 to 95 °C at a constant rate of 0.05 °C/s in a QuantStudio 6 qPCR instrument (Applied Biosystems, Singapore). Data were analyzed using the Boltzmann function. Compounds with increased melting temperature by 2 °C or more, in comparison to the control curve, were considered positives.

### Phylogenetic analyses

Amino acid sequences of *Zm*DRIK1-related plant RLK/Pelle-LRRs were retrieved from maize GDB [76], Phytozome12 [77], PLAZA 4.0 [78], TAIR [79], EnsemblPlants [80], and NCBI Databases [81] (Additional File 7: Table S2). The programs iTAK [82] and PlantsP [83] were used to annotate the kinase groups. Sequences were aligned using ClustalX [84], and the phylogenetic tree was generated with MEGA 6 [85] using the Neighbor-Joining method [86] and 1000 bootstrap replicates. The evolutionary distances were computed using the Poisson correction method [87] and were expressed as units of amino acid substitutions per site. The analyses included all *A. thaliana* and *Z. mays* subfamily LRR-VI-2 representative proteins, and orthologues of *Zm*DRIK1 from other species (Additional file 7: Table S2). The out-group sequences from Arabidopsis are represented by other subfamilies: catalytically inactive BSK8 [46], catalytically active SERK1 [88], BRI1 [89], and CLV1 [90]. The RLK/Pelle subfamily groups are classified as proposed by Lehti-Shiu et al. 2009 [8] and 2012 [91].

### Quantitative real-time PCR

Total RNA was extracted from the leaves and roots of 3- and 15-day-old B73 maize plants or leaves of drought stressed B73 maize. Samples were powdered in liquid nitrogen, and 100–200 mg powder was mixed with 1 mL of TRIzol (Invitrogen, Carlsbad, USA) and vortexed. After 10 min of incubation at room temperature, 200 μL of chloroform was added and vortexed briefly. Samples were centrifuged for 15 min at 12,000 x g at 4 °C, the aqueous phase was collected, and RNA was purified using a PureLink RNA kit (Life Technologies, Carlsbad, USA) according to the manufacturer’s instructions. After checking for RNA quality and integrity by agarose gel, cDNAs were synthesized using SuperScript III reverse transcriptase (Invitrogen, Carlsbad, USA). Five micrograms of total RNA were incubated with oligo dT20 and dNTP for 5 min at 65 °C and then cooled on ice. Buffer, DTT and SuperScript enzyme were added and incubated for 60 min at 50 °C. The enzyme was inactivated for 15 min at 70 °C, and the cDNA was stored at − 20 °C until use.

Quantitative real-time PCR (qRT-PCR) was performed in a 10 μL reaction mixture using 5 μL of 2x Luna universal qPCR master mix (New England Biolabs), 0.5 μM of each primer (Additional file 8: Table S3) and 3 μL of 20-fold diluted cDNA. qRT-PCR was performed in QuantStudio 6 (Applied Biosystems, Singapore), and the PCR conditions were as follows: 50 °C for 2 min, 95 °C for 10 min, 40 cycles of 95 °C for 15 s and 60 °C for 1 min. To analyze if single products were formed, a melting curve (starting at 60 °C and increasing by 0.05 °C/s until reaching 95 °C) at the end of the qPCR was performed. The βTUB, CYP, and EIF4a genes were used as internal controls. qRT-PCR data were analyzed using QuantStudio Real-time PCR software (version 1.3). Quantification was performed with the ΔΔCt method of three replicates and expressed as relative mRNA expression. Pairwise comparisons of mRNA level between different tissues and plant age, or between drought-stressed and rewatered samples, were calculated using a two-tailed Student’s t-test. All data analyses were conducted using GraphPad Prism version 6.01 for Windows (GraphPad Software, La Jolla, USA).

## Supplementary information

**Additional file 1: Figure S1.** Identification of *Zm*DRIK1 as a promising drought stress related receptor kinase.

**Additional file 2: Figure S2.** DRIK1 and maize RLKs present a diversified gene structure but conserved protein kinase domain.

**Additional file 3: Figure S3.***DRIK1* transcript level is downregulated by stress perturbations in maize.

**Additional file 4: Figure S4.** *DRIK1* transcript level is increased during germination in maize.

**Additional file 5: Figure S5.***Zm*DRIK1-KD bind the small molecule ENMD-2076.

**Additional file 6: Table S1.** Small molecule library thermal profiling for DRIK1 hits identification.

**Additional file 7: Table S2.** Protein IDs used for in silico analysis of *Zm*DRIK1.

**Additional file 8: Table S3.** Primers used in the RT-qPCR experiments.

**Additional file 9: Figures S6-S7.** Original SDS-PAGE gels presented in Fig. 3b.

## Data Availability

The crystallography dataset generated during the current study are available in the PDB repository (http://www.rcsb.org/structure/6CPY; http://www.rcsb.org/structure/6EAS). Other datasets used and/or analyzed during the current study are available from the corresponding author on reasonable request.

## Abbreviations

ABA: Abscisic acid
ATP: Adenosine triphosphate
DAS: Days after sowing
DSF: Differential scanning fluorimetry
GTP: Guanosine triphosphate
IEM: Interaction energy matrix
IMAC: Immobilized metal affinity chromatography
ITC: Isothermal calorimetry
KD: Kinase domain
LIC: Ligase independent cloning
LRR: Leucine-rich repeat
qRT-PCR: quantitative real-time PCR
RLK: Receptor-like kinase
Zm: *Zea mays*
