# Supplementary material for: Crystal structure of DRIK1, a stress-responsive receptor-like pseudokinase, reveals the molecular basis for the absence of ATP binding
Source: BMC Plant Biol. 2020 Apr 15;20:158. doi: 10.1186/s12870-020-2328-3 (PMC7158045; doi:10.1186/s12870-020-2328-3)
Supplement: Supplementary file 1 — Additional file 1: Figure S1. Identification of ZmDRIK1 as a promising drought stress related receptor kinase. [file 12870_2020_2328_MOESM1_ESM.pptx]

## Slide 1
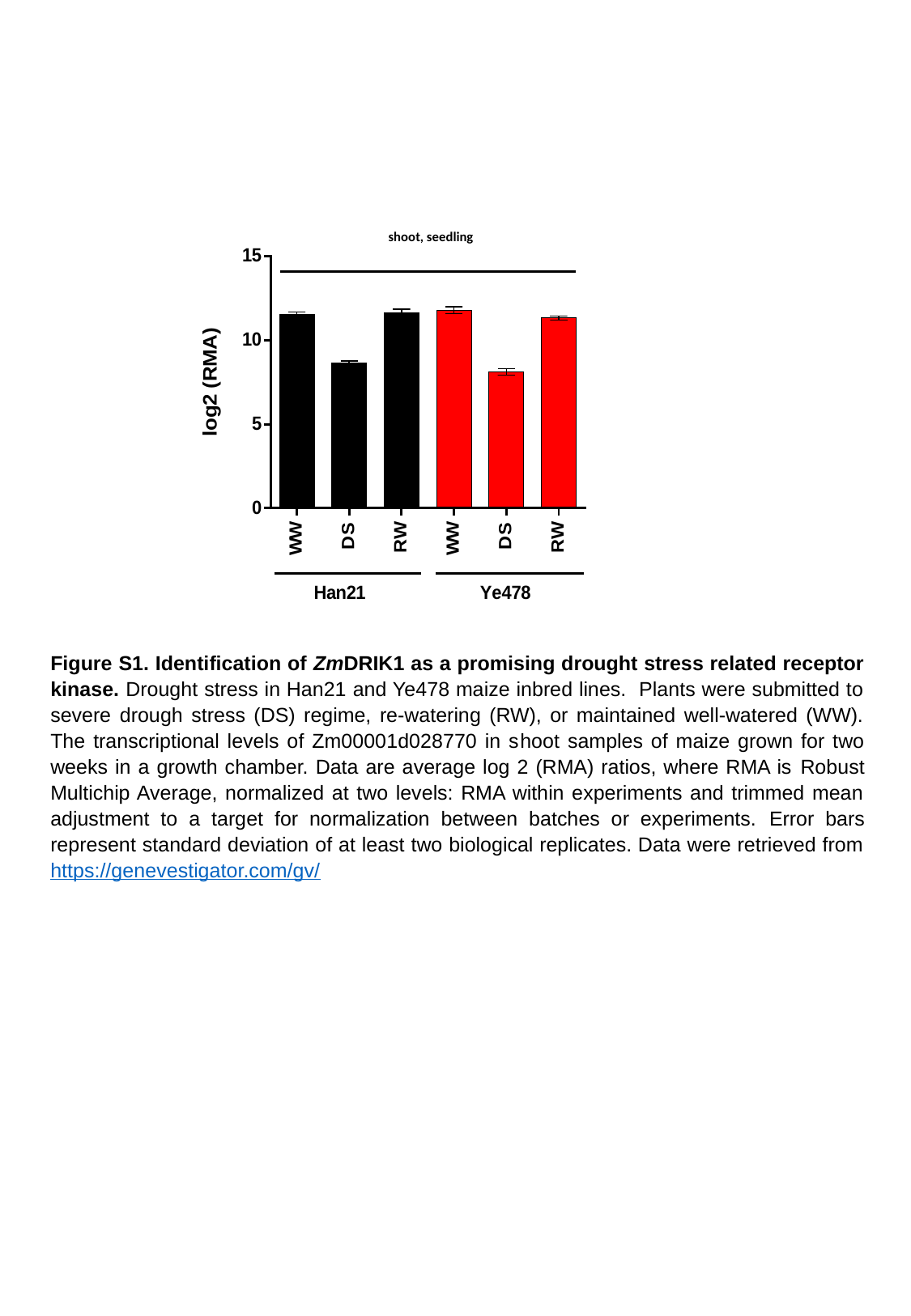

shoot, seedling
Figure S1. Identification of ZmDRIK1 as a promising drought stress related receptor kinase. Drought stress in Han21 and Ye478 maize inbred lines. Plants were submitted to severe drough stress (DS) regime, re-watering (RW), or maintained well-watered (WW). The transcriptional levels of Zm00001d028770 in shoot samples of maize grown for two weeks in a growth chamber. Data are average log 2 (RMA) ratios, where RMA is Robust Multichip Average, normalized at two levels: RMA within experiments and trimmed mean adjustment to a target for normalization between batches or experiments. Error bars represent standard deviation of at least two biological replicates. Data were retrieved from https://genevestigator.com/gv/
