# Supplementary material for: Crystal structure of DRIK1, a stress-responsive receptor-like pseudokinase, reveals the molecular basis for the absence of ATP binding
Source: BMC Plant Biol. 2020 Apr 15;20:158. doi: 10.1186/s12870-020-2328-3 (PMC7158045; doi:10.1186/s12870-020-2328-3)
Supplement: Supplementary file 2 — Additional file 2: Figure S2. DRIK1 and maize RLKs present a diversified gene structure but conserved protein kinase domain. [file 12870_2020_2328_MOESM2_ESM.pptx]

## Slide 1
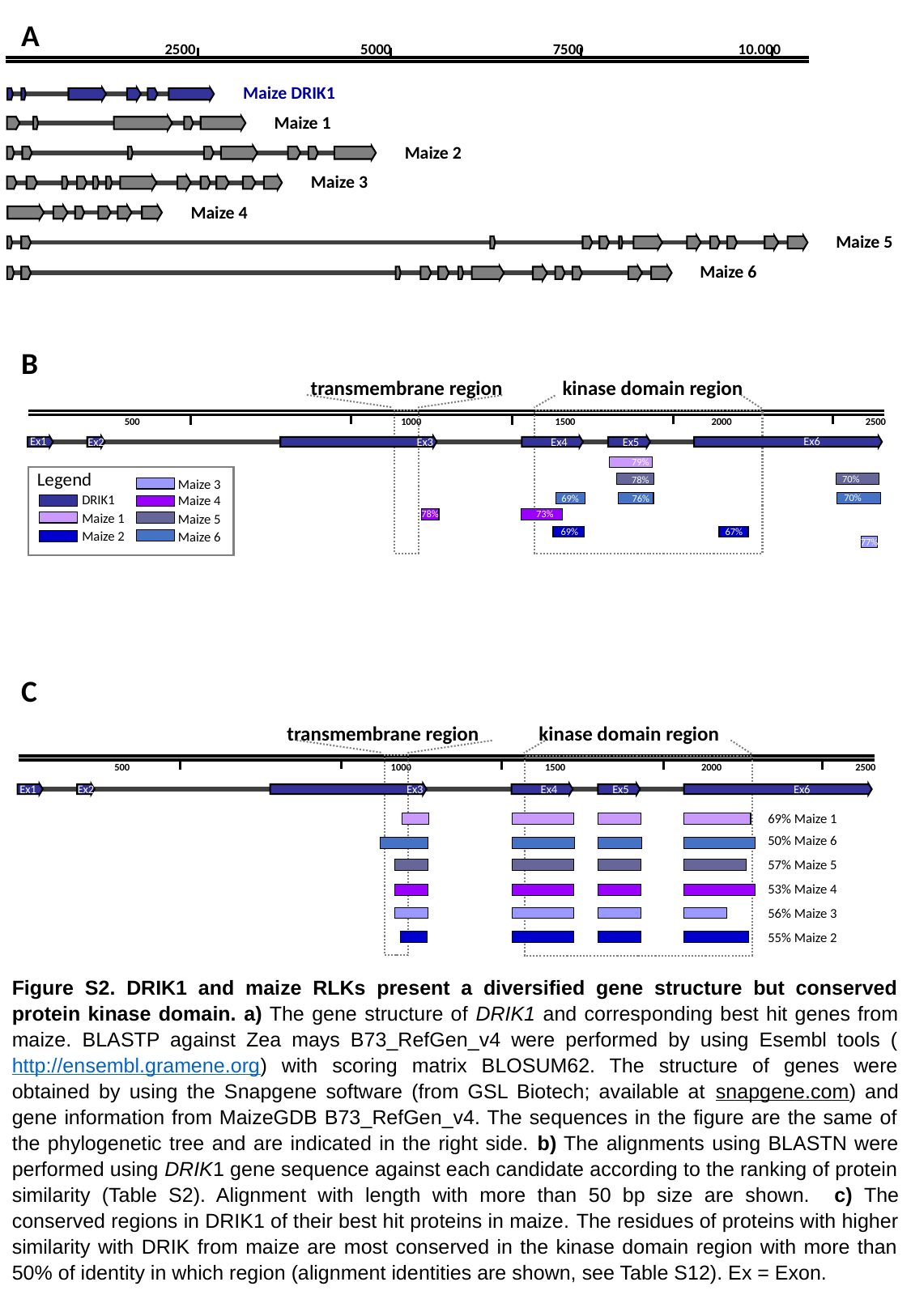

A
B
C
2500 5000 7500 10.000
Maize DRIK1
Maize 1
Maize 2
Maize 3
Maize 4
Maize 5
Maize 6
transmembrane region
kinase domain region
500		 1000 1500 2000 2500
Ex6
Ex1
Ex2
Ex3
Ex4
Ex5
79%
Legend
70%
78%
Maize 3
Maize 4
Maize 5
Maize 6
70%
DRIK1
Maize 1
Maize 2
76%
69%
78%
73%
69%
67%
77%
transmembrane region
kinase domain region
500		 1000 1500 2000 2500
Ex6
Ex1
Ex2
Ex3
Ex4
Ex5
69% Maize 1
50% Maize 6
57% Maize 5
53% Maize 4
56% Maize 3
55% Maize 2
Figure S2. DRIK1 and maize RLKs present a diversified gene structure but conserved protein kinase domain. a) The gene structure of DRIK1 and corresponding best hit genes from maize. BLASTP against Zea mays B73_RefGen_v4 were performed by using Esembl tools (http://ensembl.gramene.org) with scoring matrix BLOSUM62. The structure of genes were obtained by using the Snapgene software (from GSL Biotech; available at snapgene.com) and gene information from MaizeGDB B73_RefGen_v4. The sequences in the figure are the same of the phylogenetic tree and are indicated in the right side. b) The alignments using BLASTN were performed using DRIK1 gene sequence against each candidate according to the ranking of protein similarity (Table S2). Alignment with length with more than 50 bp size are shown. c) The conserved regions in DRIK1 of their best hit proteins in maize. The residues of proteins with higher similarity with DRIK from maize are most conserved in the kinase domain region with more than 50% of identity in which region (alignment identities are shown, see Table S12). Ex = Exon.
