# Supplementary material for: Crystal structure of DRIK1, a stress-responsive receptor-like pseudokinase, reveals the molecular basis for the absence of ATP binding
Source: BMC Plant Biol. 2020 Apr 15;20:158. doi: 10.1186/s12870-020-2328-3 (PMC7158045; doi:10.1186/s12870-020-2328-3)
Supplement: Supplementary file 3 — Additional file 3: Figure S3.DRIK1 transcript level is downregulated by stress perturbations in maize. [file 12870_2020_2328_MOESM3_ESM.pptx]

## Slide 1
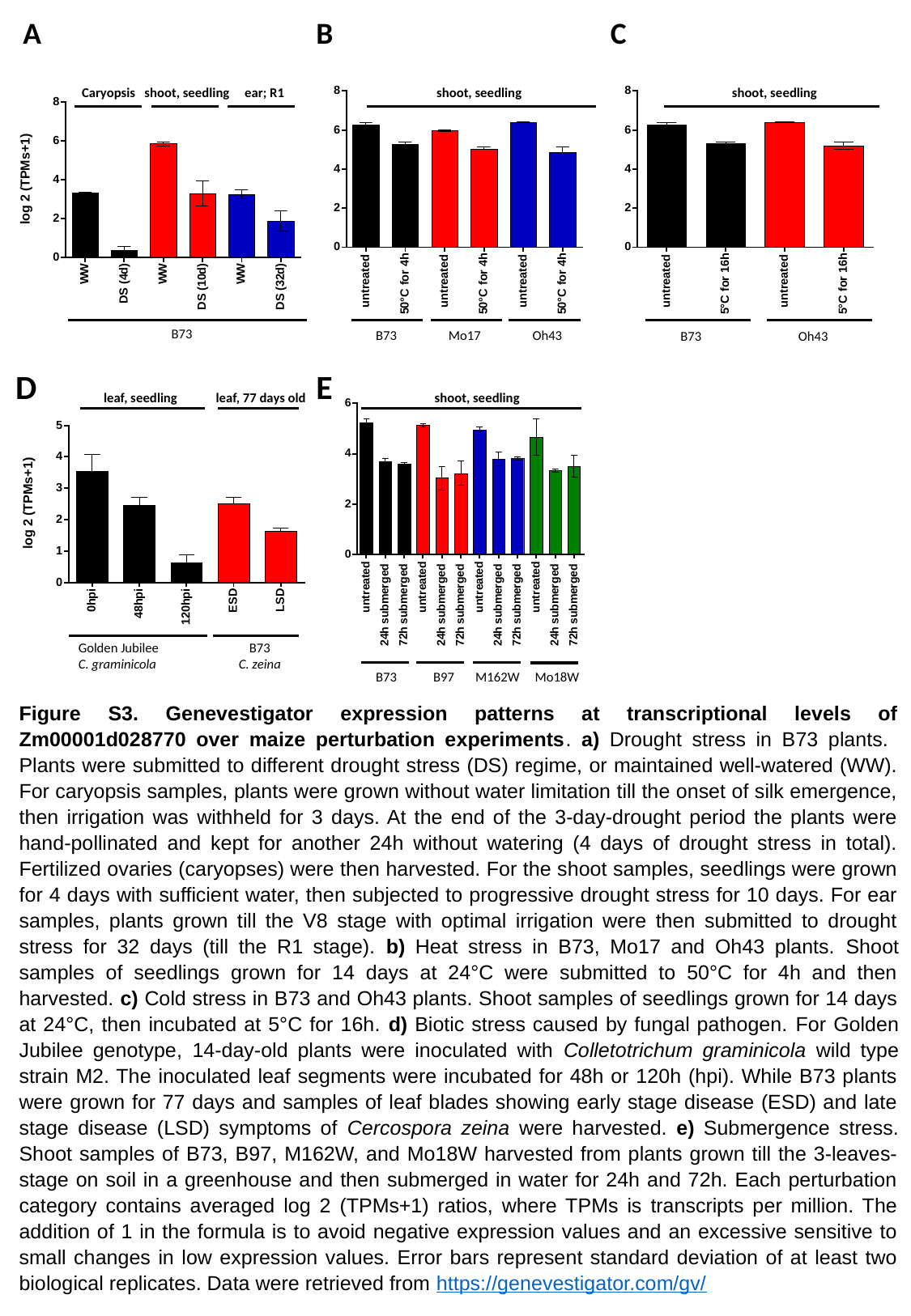

A
B
C
Caryopsis shoot, seedling ear; R1
shoot, seedling
shoot, seedling
 B73
 B73 Mo17 Oh43
 B73 Oh43
D
E
leaf, seedling
leaf, 77 days old
shoot, seedling
Golden Jubilee
C. graminicola
B73
 C. zeina
B73 B97 M162W Mo18W
Figure S3. Genevestigator expression patterns at transcriptional levels of Zm00001d028770 over maize perturbation experiments. a) Drought stress in B73 plants. Plants were submitted to different drought stress (DS) regime, or maintained well-watered (WW). For caryopsis samples, plants were grown without water limitation till the onset of silk emergence, then irrigation was withheld for 3 days. At the end of the 3-day-drought period the plants were hand-pollinated and kept for another 24h without watering (4 days of drought stress in total). Fertilized ovaries (caryopses) were then harvested. For the shoot samples, seedlings were grown for 4 days with sufficient water, then subjected to progressive drought stress for 10 days. For ear samples, plants grown till the V8 stage with optimal irrigation were then submitted to drought stress for 32 days (till the R1 stage). b) Heat stress in B73, Mo17 and Oh43 plants. Shoot samples of seedlings grown for 14 days at 24°C were submitted to 50°C for 4h and then harvested. c) Cold stress in B73 and Oh43 plants. Shoot samples of seedlings grown for 14 days at 24°C, then incubated at 5°C for 16h. d) Biotic stress caused by fungal pathogen. For Golden Jubilee genotype, 14-day-old plants were inoculated with Colletotrichum graminicola wild type strain M2. The inoculated leaf segments were incubated for 48h or 120h (hpi). While B73 plants were grown for 77 days and samples of leaf blades showing early stage disease (ESD) and late stage disease (LSD) symptoms of Cercospora zeina were harvested. e) Submergence stress. Shoot samples of B73, B97, M162W, and Mo18W harvested from plants grown till the 3-leaves-stage on soil in a greenhouse and then submerged in water for 24h and 72h. Each perturbation category contains averaged log 2 (TPMs+1) ratios, where TPMs is transcripts per million. The addition of 1 in the formula is to avoid negative expression values and an excessive sensitive to small changes in low expression values. Error bars represent standard deviation of at least two biological replicates. Data were retrieved from https://genevestigator.com/gv/
