# Supplementary material for: Crystal structure of DRIK1, a stress-responsive receptor-like pseudokinase, reveals the molecular basis for the absence of ATP binding
Source: BMC Plant Biol. 2020 Apr 15;20:158. doi: 10.1186/s12870-020-2328-3 (PMC7158045; doi:10.1186/s12870-020-2328-3)
Supplement: Supplementary file 4 — Additional file 4: Figure S4. DRIK1 transcript level is increased during germination in maize. [file 12870_2020_2328_MOESM4_ESM.pptx]

## Slide 1
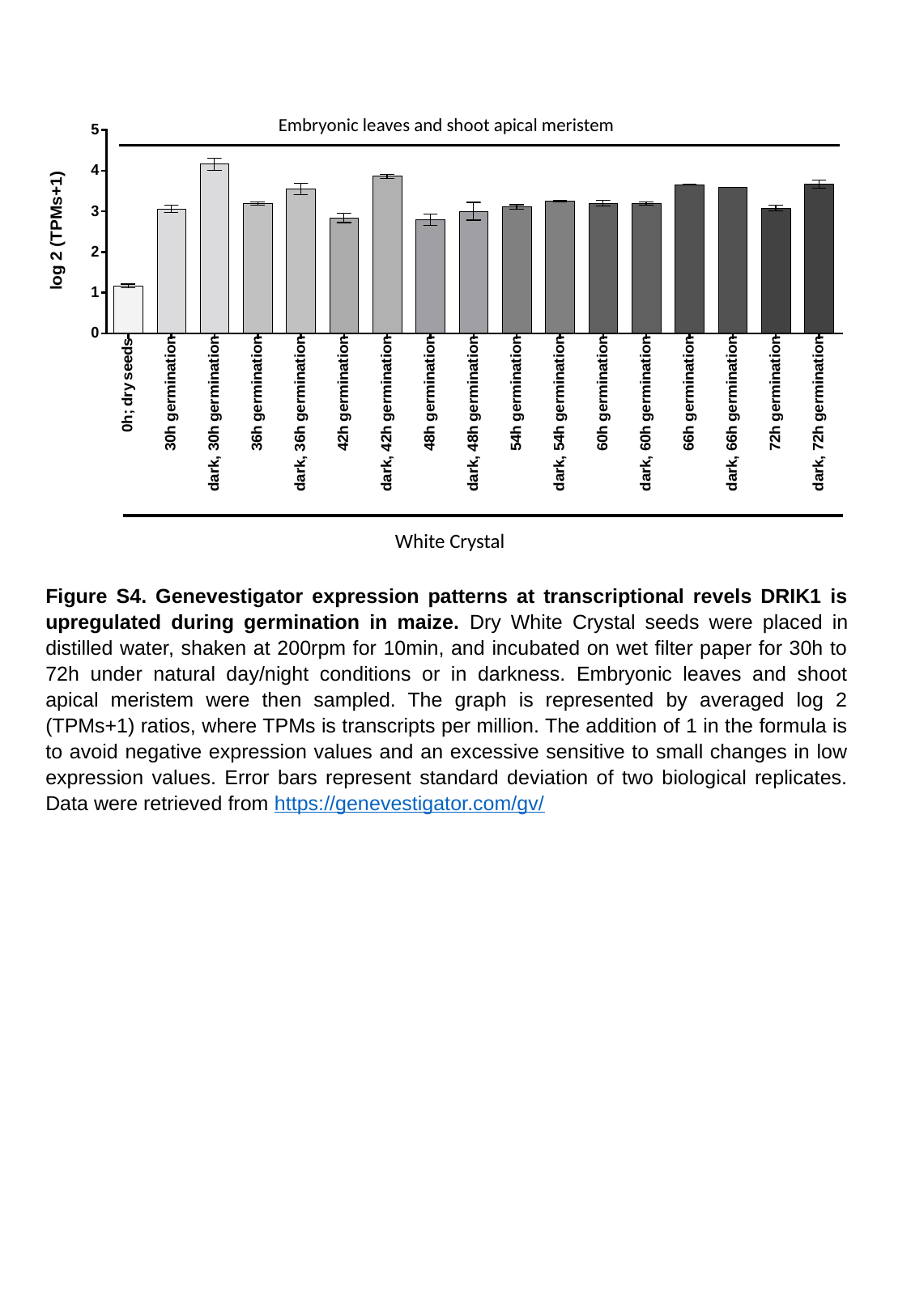

Embryonic leaves and shoot apical meristem
 White Crystal
Figure S4. Genevestigator expression patterns at transcriptional revels DRIK1 is upregulated during germination in maize. Dry White Crystal seeds were placed in distilled water, shaken at 200rpm for 10min, and incubated on wet filter paper for 30h to 72h under natural day/night conditions or in darkness. Embryonic leaves and shoot apical meristem were then sampled. The graph is represented by averaged log 2 (TPMs+1) ratios, where TPMs is transcripts per million. The addition of 1 in the formula is to avoid negative expression values and an excessive sensitive to small changes in low expression values. Error bars represent standard deviation of two biological replicates. Data were retrieved from https://genevestigator.com/gv/
