# Supplementary material for: Crystal structure of DRIK1, a stress-responsive receptor-like pseudokinase, reveals the molecular basis for the absence of ATP binding
Source: BMC Plant Biol. 2020 Apr 15;20:158. doi: 10.1186/s12870-020-2328-3 (PMC7158045; doi:10.1186/s12870-020-2328-3)
Supplement: Supplementary file 5 — Additional file 5: Figure S5.ZmDRIK1-KD bind the small molecule ENMD-2076. [file 12870_2020_2328_MOESM5_ESM.pptx]

## Slide 1
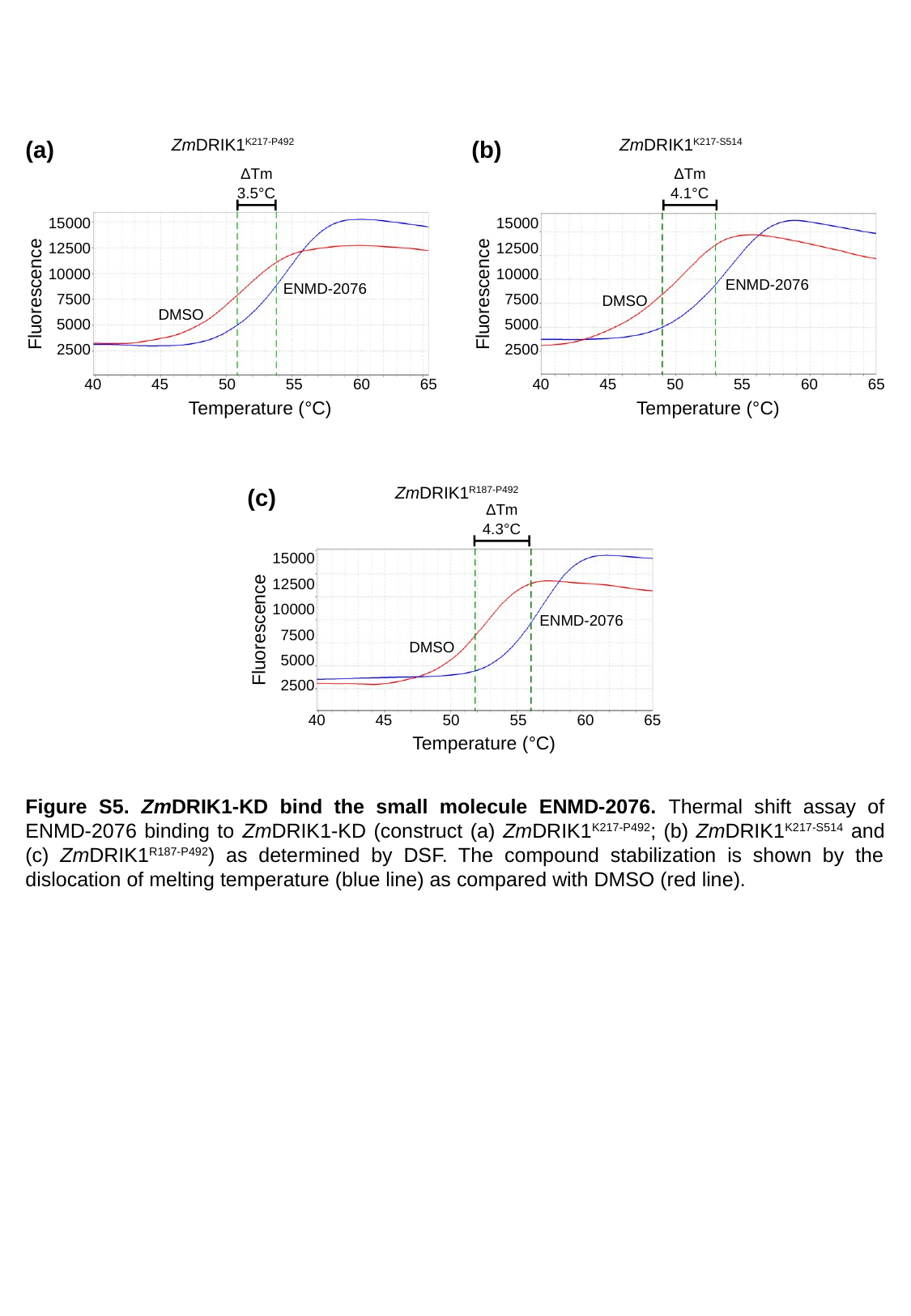

(a)
ZmDRIK1K217-P492
(b)
ZmDRIK1K217-S514
ΔTm
3.5°C
15000
12500
10000
ENMD-2076
Fluorescence
7500
DMSO
5000
2500
40
45
50
55
60
65
Temperature (°C)
ΔTm
4.1°C
15000
12500
10000
ENMD-2076
Fluorescence
7500
DMSO
5000
2500
40
45
50
55
60
65
Temperature (°C)
(c)
ZmDRIK1R187-P492
ΔTm
4.3°C
15000
12500
10000
ENMD-2076
Fluorescence
7500
DMSO
5000
2500
40
45
50
55
60
65
Temperature (°C)
Figure S5. ZmDRIK1-KD bind the small molecule ENMD-2076. Thermal shift assay of ENMD-2076 binding to ZmDRIK1-KD (construct (a) ZmDRIK1K217-P492; (b) ZmDRIK1K217-S514 and (c) ZmDRIK1R187-P492) as determined by DSF. The compound stabilization is shown by the dislocation of melting temperature (blue line) as compared with DMSO (red line).
