# Supplementary material for: Crystal structure of DRIK1, a stress-responsive receptor-like pseudokinase, reveals the molecular basis for the absence of ATP binding
Source: BMC Plant Biol. 2020 Apr 15;20:158. doi: 10.1186/s12870-020-2328-3 (PMC7158045; doi:10.1186/s12870-020-2328-3)
Supplement: Supplementary file 7 — Additional file 7: Table S2. Protein IDs used for in silico analysis of ZmDRIK1. [file 12870_2020_2328_MOESM7_ESM.docx]

**Table S2. Protein IDs used for *in silico* analysis of *Zm*DRIK1**

| Organism */*  Abreviation | ID and Database | Organism */*  Abreviation | ID and Database |
| --- | --- | --- | --- |
| *Zea mays /*  DRIK1 | Zm00001d028770_P001 (maizeGDB) XP_008667250.1 (NCBI) | *Setaria italica* */* Foxtail_millet | Seita.9G439200.1 (Phytozome12)  XP_004984869.1 (NCBI) |
| *Zea mays /*  Maize_1 | Zm00001d038020_P01 (maizeGDB)  NP_001350868.1 (NCBI) | *Triticum urartu /* Wheat | TRIUR3_19380-T1 (EnsemblPlants)  EMS47067.1 (NCBI) |
| *Zea mays* */*  Maize_2 | Zm00001d038432_P06 (maizeGDB)  XP_008649735.1 (NCBI) | *Arabidopsis thaliana* */* Arabidopsis_1 | At5g07150 (TAIR)  EFH47512.1 (NCBI) |
| *Zea mays /*  Maize_3 | Zm00001d039406_P01 (maizeGDB)  NP_001310964.1 (NCBI) | *Arabidopsis thaliana* */* Arabidopsis_2 | At5g58540  XP_020886868.1 (NCBI) |
| *Zea mays* */*  Maize_4 | Zm00001d038991_P001(maizeGDB)  NP_001136868.1 (NCBI) | *Arabidopsis thaliana* */* Arabidopsis_3 | At3g56050 (TAIR)  XP_020880897.1 (NCBI) |
| *Zea mays* */*  Maize_5 | Zm00001d011580_P001 (maizeGDB)  NP_001130369.1 (NCBI) | *Arabidopsis thaliana* */* Arabidopsis_4 | At2g40270.1 (TAIR)  NP_565925.1 (NCBI) |
| *Zea mays* */*  Maize_6 | Zm00001d043890_P002 (maizeGDB)  XP_008675203.1 (NCBI) | *Arabidopsis thaliana* */* Arabidopsis_5 | At4g18640 (TAIR)  NP_193599.3 (NCBI) |
| *Brachypodium distachyon /* Brachypodium | BRADI1g65340v3 (EnsemblPlants) XP_003558141.1 (NCBI) | *Arabidopsis thaliana* */* Arabidopsis_6 | At1g63430.1 (TAIR)  NP_176532.2 (NCBI) |
| *Panicum hallii /* Panicgrass | Pahal_I04411 (Phytozome 12)  XP_025791728.1 (NCBI) | *Arabidopsis thaliana* */* Arabidopsis_7 | At5g41180.1 (TAIR)  NP_198934.1 (NCBI) |
| *Oropetium thomaeum* */* Oropetium | Oropetium_20150105_01022A (PLAZA 4.0) | *Arabidopsis thaliana* */* AtBSK8 | At5g41260 (TAIR)  NP_198942.1 (NCBI) |
| *Orysa sativa* */* Rice | LOC_Os03g18370.1  XP_015628739.1 (NCBI) | *Arabidopsis thaliana* */* AtCLV1 | At1g75820 (TAIR)  NP_177710.1 (NCBI) |
| *Glycine max* */* Soybean | Glyma.11G167100.1 (PLAZA 4.0)  XP_006591246.1 (NCBI) | *Arabidopsis thaliana* */* AtSERK1 | At1g71830.1 (TAIR)  NP_177328.1 (NCBI) |
| *Hordeum vulgare* */* Barley | HORVU4Hr1G055890.2 (EnsemblPlants) BAJ91375.1 (NCBI) | *Arabidopsis thaliana* */* AtBRI1 | At4g39400.1 (TAIR)  NP_195650.1 (NCBI) |
| *Sorghum bicolor* */* Sorghum | SORBI_3001G405000 (EnsemblPlants)  XP_021308672.1 (NCBI) |  |  |
