# Supplementary material for: Crystal structure of DRIK1, a stress-responsive receptor-like pseudokinase, reveals the molecular basis for the absence of ATP binding
Source: BMC Plant Biol. 2020 Apr 15;20:158. doi: 10.1186/s12870-020-2328-3 (PMC7158045; doi:10.1186/s12870-020-2328-3)
Supplement: Supplementary file 8 — Additional file 8: Table S3. Primers used in the RT-qPCR experiments. [file 12870_2020_2328_MOESM8_ESM.docx]

**Table S3. Primers used in the RT-qPCR experiment**

| Gene | Accession Number | Primer Sequence (5’-3’) - Forward | Primer Sequence (5’-3’) - Reverse |
| --- | --- | --- | --- |
| β-tubulin | NP_001105457 | CTACCTCACGGCATCTGCTATGT | GTCACACACACTCGACTTCACG |
| EIF4a | NM_001111902 | CGTCCAGAGGTTCTACAA | CATCCTTCGCCACAATAC |
| ZmDRIK1 | NM_001136924.1 | CTCGGGAGCTCAGGGATTG | GTGAGAGCCCGTGTATGTGG |
