# Supplementary material for: Crystal structure of DRIK1, a stress-responsive receptor-like pseudokinase, reveals the molecular basis for the absence of ATP binding
Source: BMC Plant Biol. 2020 Apr 15;20:158. doi: 10.1186/s12870-020-2328-3 (PMC7158045; doi:10.1186/s12870-020-2328-3)
Supplement: Supplementary file 9 — Additional file 9: Figures S6-S7. Original SDS-PAGE gels presented in Fig. 3b. [file 12870_2020_2328_MOESM9_ESM.pptx]

## Slide 1
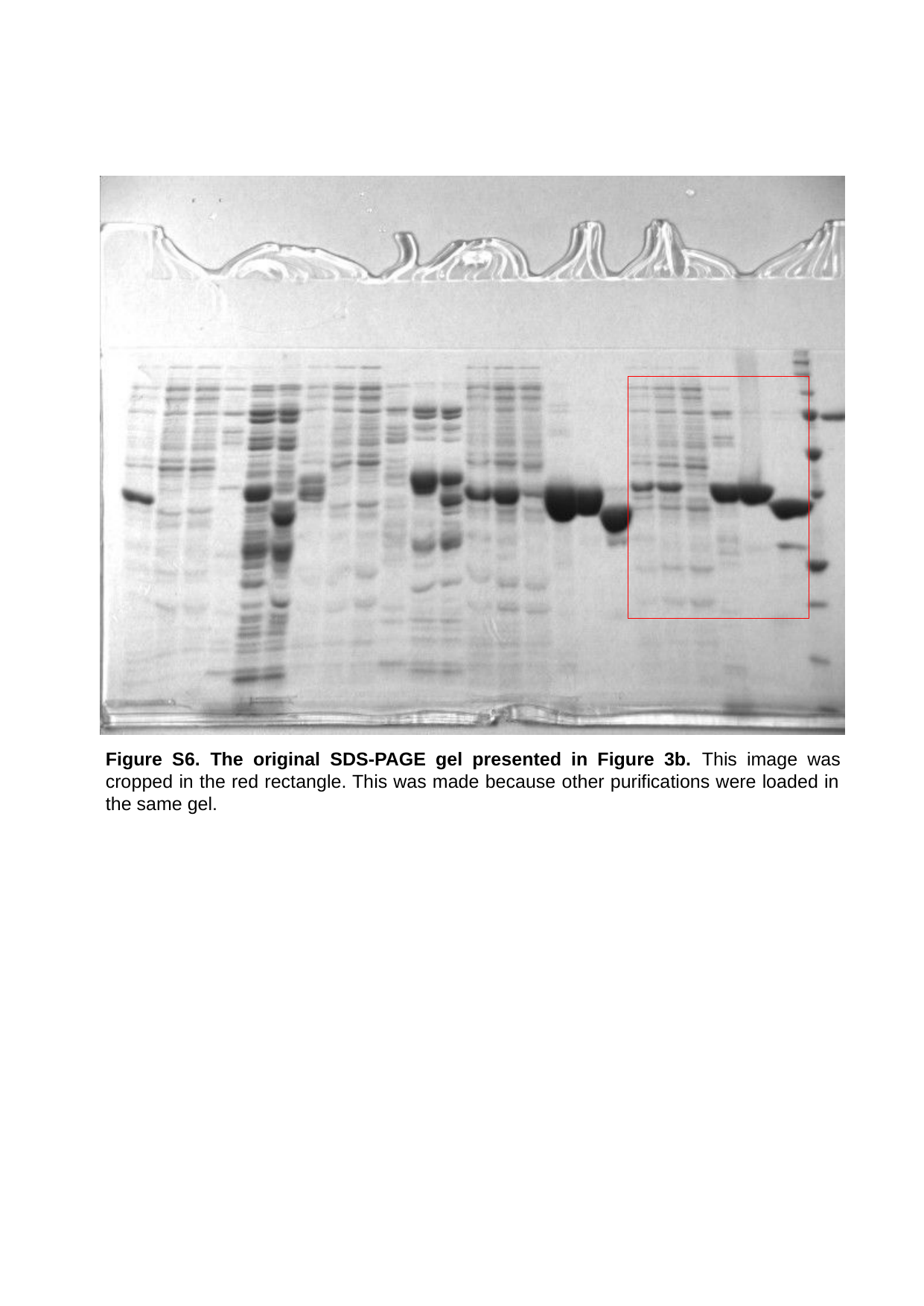

Figure S6. The original SDS-PAGE gel presented in Figure 3b. This image was cropped in the red rectangle. This was made because other purifications were loaded in the same gel.

## Slide 2
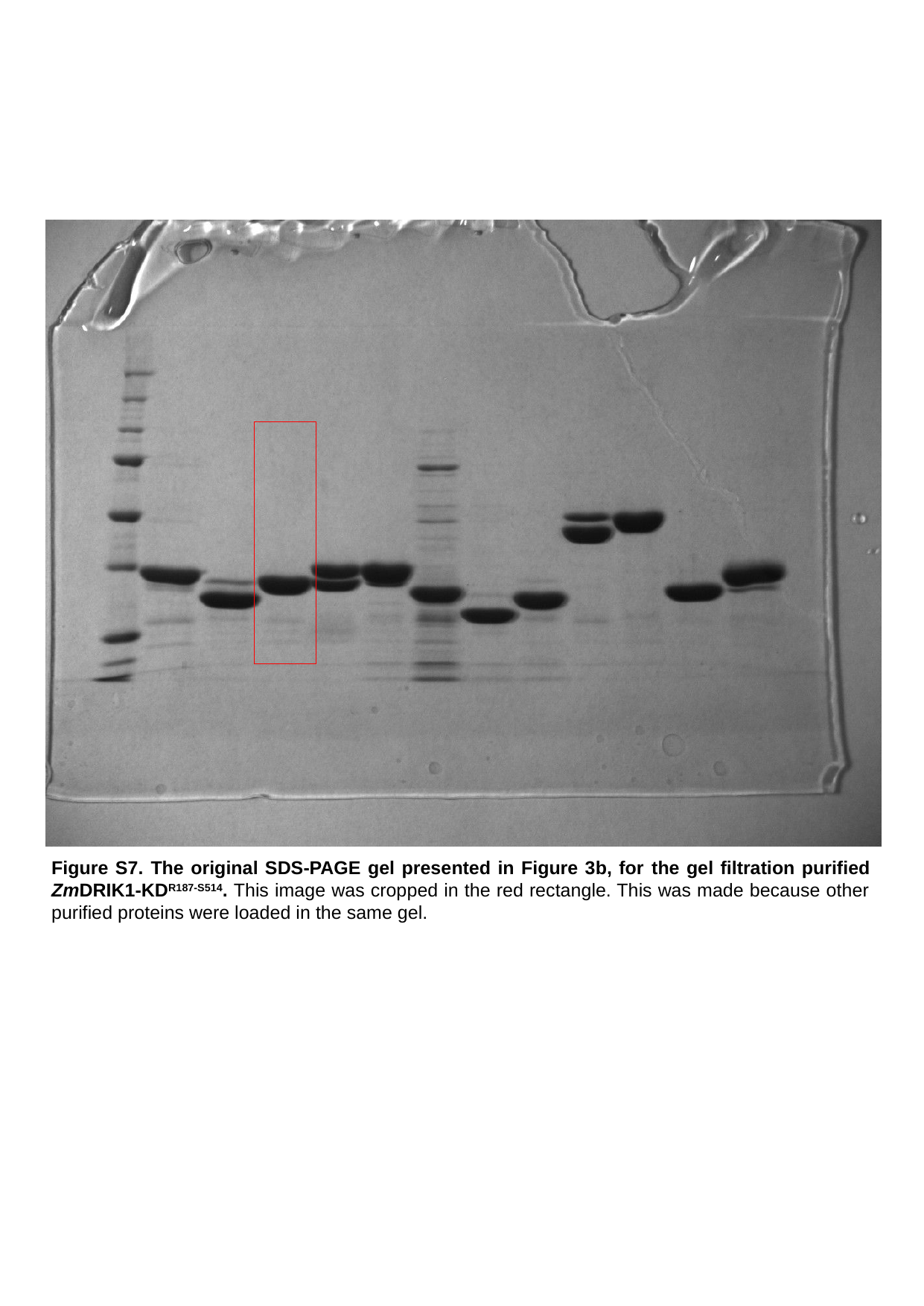

Figure S7. The original SDS-PAGE gel presented in Figure 3b, for the gel filtration purified ZmDRIK1-KDR187-S514. This image was cropped in the red rectangle. This was made because other purified proteins were loaded in the same gel.
